# Supplementary material for: Malaria outbreak facilitated by engagement in activities near swamps following increased rainfall and limited preventive measures: Oyam District, Uganda
Source: PLOS Glob Public Health. 2022 Aug 23;2(8):e0000239. doi: 10.1371/journal.pgph.0000239 (PMC10021189; doi:10.1371/journal.pgph.0000239)
Supplement: Attachment — Submitted filename: Response to Reviewers.doc [file pgph.0000239.s001.doc]

**January 31, 2022**

The Editor-in-Chief

*PLOS Global Public Health*

Dear Editor,

**Re: Revised Manuscript: “Malaria Outbreak Facilitated by Engagement in Activities near Swamps Following Increased Rainfall and Limited Preventive Measures: Oyam District, Uganda, PGPH-D-21-00604”**

We are grateful for the opportunity to revise our manuscript entitled, “**Malaria Outbreak Facilitated by Engagement in Activities near Swamps Following Increased Rainfall and Limited Preventive Measures: Oyam District, Uganda**”. The reviewers’ comments were very helpful in improving the paper in a substantial manner.

On the next page, we present a point-by-point response to each of the comments raised. I should note that in the process of revising the paper, we were able to make adjustments and improvements across the paper to ensure that the paper is generally improved for your readers.

As advised, we have submitted three documents: a) a clean copy of the revised manuscript; b) a copy of the revised manuscript with track changes; and c) a point-by-point response to the comments raised (see page 2 below).

Once again, we are very grateful for the opportunity to revise the paper and we look forward to seeing the paper accepted and eventually published in your prestigious journal, PLOS Global Public Health.

Yours sincerely,

Maureen Katusiime

**Corresponding author**

**POINT-BY-POINT RESPONSE TO REVIEWERS’ COMMENTS**

| **No** | **Comment** | **Response** |
| --- | --- | --- |
|  | **Journal Requirements** |  |
|  | **Methods** |  |
| 2 | Please clarify that participants provided oral consent. Please also state in the methods why written consent could not be obtained, how oral consent was documented | We thank the reviewer for raising this suggestion. We have included the Ethical consideration and consent to participate in the study under the methods section. We have addressed on line 271 to 277. |
| 3 | In your methods section, please provide additional information regarding the permits you obtained for the work. Please ensure you have included the full name of the authority that approved the field site access and if no permits were required, a brief statement explaining why | We have addressed this comment. Please see line 264-271 under Ethical consideration and consent to participate |
| 4 | Please provide separate figure files in tif or eps format only, and remove any figures embedded in your manuscript file. If you are using Latex, you need to remove embedded figures. | We have provided separate figure files in tif as guided |
| 5 | You have uploaded supporting information but you have not included a list of legends. Please add a full list of legends for all supporting information files (including figures, table and data files) after the reference list. | I have included a list of legends for all supporting files after the reference list |
| 6 | Please explain via email why the data is not available. Please also include the contact information for the third party organization that should be contacted should other researchers want to request access to this data and please include the full citation of where the data can be found. We also request that you verify with us via email that any researcher will be able to obtain the data set in the same manner that the you have obtained it. If you feel you are unwilling or unable to adhere to this policy, please explain your reasons by return email and your exemption request will be escalated to the editor for approval. Your exemption request will be handled independently and will not hold up the peer review process, but will need to be resolved should your manuscript be accepted for publication. One of the Editorial team will be in touch if they require more information. | Thank you for this guidance. I have sent an email in this regard highlighting that : “ The datasets upon which our findings are based belong to the Uganda Public Health Fellowship Program. For confidentiality reasons, the datasets are not publicly available. However, the data sets can be availed upon reasonable request from the corresponding author and with permission from the Uganda Public Health Fellowship Program.”  Contact information [mkatusiime@musph.ac.ug](mailto:mkatusiime@musph.ac.ug) |
| 7 | Please provide us with a direct link to the base layer of the map used in figure 2 and figure 4 and ensure this location is also included in the figure legend | The map in figure 2 and figure 4 was drawn using qgis version 3.2. The baselayer for the map used in Figure 4 was obtained from open street map within qgis version 3.2. qgis is an open source software or open license. Within qgis, we linked subcounty shape files from Uganda Bureau of Statistics (UBOS) to the base layer. We have included two links one for the open street map and the other on how to link shape files using vector files in qgis.  Figure 2 was simply drawn using qgis and subcounty shape files from UBOS. |
|  | **Reviewer 1** |  |
| 8 | Malaria outbreak investigation is an important topic in global public health especially in endemic countries. The authors did a case control study to investigate the scope of the outbreak, identified exposures associated with increased transmission, and recommended evidence-based control measures. However, the paper does not flow especially in the methods section. | We think this is an important observation that was raised by the reviewer. We have modified the methods section so that it flows. However, we organized the method section to follow the standard steps of an outbreak investigation since the findings are typically from an outbreak investigation. |
| 9 | The authors also did entomological monitoring but missed out important parameters such as sporozoite rates and entomological inoculation rates. The mosquito house resting density and mosquito age parameters measured were not interpreted. For example, If 65% of the mosquitoes collected were blood fed, what this mean on bed net use? | We think this is an important observation that was raised by the reviewer. However, we have highlighted these parameters among the limitations of the study because it was beyond the scope of this investigation. |
|  | **Abstract** |  |
| 10 | Line 29-30 in the statement “We conducted entomological and environmental assessments.”. Elaborate why this was done like you did in line 30-31 in the statement “We interviewed 83 case-patients and 83 asymptomatic age- and parish-matched controls to determine exposures associated with illness” | We have taken note of this important comment. We have addressed it in line 31-32 of the abstract. |
| 11 | Change breeding sites to Anopheles aquatic habitats | We have changed breeding sites to Anopheles aquatic habitats in the whole document. |
| 12 | For the statement “sand pits (25/350mls) and brick pits (16/350mls) had the highest average larval concentrations” which is not clear. If you aimed at quantifying larval densities, you would measure the number of larvae per scoop. | We thank you for this comment. This actually meant 25 larvae per scoop taken from sandpits and 16 larvae per scoop.  We used a standard dipper (350mls) as recommended by WHO. |
|  | Methods |  |
| 13 | You need to further describe the study area including economic activities in the area. | We have addressed this comment in the methods section on line 112-114 |
| 14 | You need not clarify the health service delivery in the sub-county; How many health units are in the sub-county, the levels of these health units, how many were visited. | We have addressed the health service delivery in the sub-country and the number in line 115.  We visited all Health facilities as addressed under the section of case definition and finding on line 122-123 |
| 15 | The two statements from line 95-98 sound the same. In line 120, is 2km a radius? This normally done using transects of given width and length | We have modified the writing. We have deleted the line highlighting the 2km radius because we estimated it visually and was not systematically done . We have acknowledged it as a limitation under the limitation section of the manuscript on line 478-481, on page 23 |
| 16 | You will need to define all aquatic habitats (what you call breeding sites). | Thank you for this suggestion. We have modified this across the whole document to reflect aquatic habitats |
| 17 | I did not see how rainfall data was obtained. | We thank you for this observation. This has been addressed under the section of Rainfall on line 165-169 |
| 18 | In line 126, change the word trapped to collected | We have changed the word from trapped to collected on line 176 |
| 19 | Inline 130, name the arerosol used. | This has been addressed on line 180-181 |
| 20 | In line 131, give a reference of the identification key used | This has been addressed on line 183 |
| 21 | Petri dishes should be changed to petri dishes (From upper case) | We have changed as suggested |
| 22 | In line 133, you mean mosquitoes were dissected, ovaries dried before examining them. | No we examined the mosquitoes for morphological features. Then after examined the dried ovaries to determine parity rate. |
| 23 | Entomological assessment should be broken done to Adult mosquito collection and Mosquito larval survey. | This has been addressed as shown in line 175 and 210 |
| 24 | Line 152-154, the word “stagnant water”, see my comment on breeding site. | Modified accordingly across the whole document to reflect anopheles aquatic habitats |
| 25 | You mixed environmental assessment and larval survey (Make the method flow logically) | Modified accordingly as guided by separating environmental assessment on line 147 from entomological assessment on line 172 |
|  | **Reviewer 2** |  |
| 26 | There are several references provided that are rather strange or unavailable for instance, 2 and 4, or even repeated under a different listing (for instance, 4 and 37 appear to be the same). Please consider updating the citation using a proper reference manager | We think this is an important observation that was raised by the reviewer. We have updated our reference list as removed some references. |
| 27 | Lines 69-70: It’s not clear what timepoint the problems discussed are referring to. They all seem to be from well before the present study duration, as indicated by the references provided. Please consider providing more relevant evidence for these or more current potential justifications for the outbreak. | We have updated the references by getting more recent ones and deleting the old ones. |
| 28 | For instance, it’s unlikely inappropriate case management was the major driver, given the current high testing rates – Please also provide a definition for in/appropriate case management. | According to WHO (<https://www.ncbi.nlm.nih.gov/books/NBK568494/>)  Malaria [case management](https://www.ncbi.nlm.nih.gov/books/n/who339609/ch10/def-item/gl1_DL1_DI40/), consists of early [diagnosis](https://www.ncbi.nlm.nih.gov/books/n/who339609/ch10/def-item/gl1_DL1_DI56/) and prompt effective treatment, remains a vital component of [malaria control](https://www.ncbi.nlm.nih.gov/books/n/who339609/ch10/def-item/gl1_DL1_DI131/) and elimination strategies.  Case management can be affected by the inefficient supply chain management of malaria commodities such as test kits, treatment drugs which is a critical challenge as highlighted in the Uganda Malaria Reduction and Elimination Strategic Plan 2021-2025 (URMP)  So inappropriate case management can be a driver even with high testing rates if people cannot access prompt effective treatment.  On line 79-83, In have included the challenge of inefficient supply chain management of malaria commodities which compromises appropriate case management as highlighted in the UMRP |
| 29 | It may also not be weak surveillance, given the existence of what’s known as integrated disease surveillance and response (IDSR) – Please consider reviewing IDSR and supporting your argument accordingly. | Thank you for this comment. I have modified the writing to focus on malaria surveillance system on line 83-87.  Yes the existence of IDSR does not necessarily translate into an adequate malaria surveillance system. I have reviewed the URMP 2021-2025 and it does highlight that the National Malaria Control Program continues to face a challenge pf inadequate use of malaria surveillance data. It further highlights epidemic prevention, preparedness and response as a core intervention and also highlighted in the strategic shifts for the plan.  Also the end term review of the Uganda Malaria Reduction Strategic Plan 2014-2020 found that the malaria epidemic preparedness and response in the country was reactive rather than proactive.  A surveillance system can only be adequate is data is collected, collated, analyzed and used to inform decisions or practice. If there is a loophole along the cycle, then that system is inadequate. |
| 30 | Line 73 – Here you confirm the effectiveness of surveillance with indication that districts were able to detect the upsurge and reported it. Is this not conflicting with your earlier assertions? | Thank you for this comment. We have modified the writing accordingly using more recent literature |
| 31 | Line 80 – you suggest that you are going to recommend evidence-based control measures. However, this upsurge was in 2019, would this be appropriate for 2021? | Thank you for this comment.  This investigation was conducted in 2019 to find factors associated with the increased transmission. We used these findings to guide the district to control the outbreak in 2019.  In line 98, we have added the time component of 2019 when this investigation was conducted. |
| 32 | Line # 84-85: It is not entirely clear what you meant by the “wet season” that lasts 11 months of the year. Please provide some clarification on this Line #86: States that the “dry season is hot and mostly cloudy”, which part of Uganda would this be whose dry season takes this description? Altogether, it appears like the calendar year is comprised of 13 months, which is rather confusing (11 dry and 2 wet!?) Lines 84-89: You describe 4 seasons including: wet, dry, hot, and cool. Please simplify or make clearer which is which? | Thank you for this observation. We have modified the write up to make it clearer as reflected on line 106 to 110. We have modified to reflect 12 months and to show wet and dry seasons. |
| 33 | Line 94-95 – It’s not clear what you mean by “increasing specificity associated with cases” were you assessing specificity? | We have deleted the statement to avoid confusion |
| 34 | Line 97 – Among the data sets you were collecting was “date of onset of clinical symptoms”. Would you please provide some indication of availability and completeness of this particular data variable in HMIS? | We have taken note of this important comment. We have deleted date of onset because this was a typing error. It was only captured during the interviews not during data abstraction from outpatient registers |
| 35 | Line 114 – It’s not clear whether the assumption here is that village and parish population estimates remained the same between 2014 when the census was conducted and 2019 when data for this study was collected. Why were population estimates of 2014 used in 2019? | We thank the reviewer for raising this comment. We have modified the write up as reflected in line 142-143. We used population estimates for 2019 from the Uganda population and Housing Census data for 2014, projected to 2019. |
| 36 | Line 119 – Please clarify what you mean by “physical features” are these hills, valleys, buildings, …? | We modified the write up and deleted physical features. We replaced with human factors and gave examples such as construction sites, soil mining sites, brick making as shown from line 148 to 155 on page 7 under the section of Environmental assessment |
| 37 | Line 120 – What approach was used to determine the 2Km distance that was used? | We used a visual scanning which is not very systematic approach. We have deleted the 2 km radius statement. We have highlighted it as a limitation for the study line 478-481 |
| 38 | Line 165-166 – It’s not clear what the sample of 20 confirmed case-persons were interviewed to do?  This paragraph seems to jump from one thing to another and leaves the reader wondering what it’s leading up to | Thank you for this comment. The 20 confirmed case-persons were interviewed to develop a feasible working hypothesis on possible contributors to the malaria outbreak. This has been included under hypothesis generation in line 221-222.  Hypothesis generation is a critical step in an outbreak investigation before conducting an analytical study in this case the case control study. |
| 39 | Line 178-179 – Given your definition of exposure as being based on fever, it’s not clear how you determined controls that were exposed. Please clarify | Thank you for this observation. We have deleted the statement on fever and modified the write-up accordingly as shown in line 228-229. |
| 40 | Line 179 – Sounds confusing that you measured “radius of a river”. How did you do this? | We used a visual scanning which is not very systematic approach. We have deleted the 2 km radius statement. We have highlighted it as a limitation for the study line 478-481 |
| 41 | Lines 198-199 – Given no mention of rainfall data till this point, how did you use the t-test to determine rainfall differences between years? | We have addressed this comment on page 8, line 167 to 169 |
| 42 | Line 202: Please consider using a more suitable title, it is not clear what you mean by “descriptive epidemiology” | Thank you for this comment. Descriptive Epidemiology is a standard term used in epidemiology to describe the person, place and time characteristics of the population being investigated or studied.  This term is used a lot in outbreak investigations and we may not modify the title because it is a standard term. |
| 43 | Figure 1 has no key, making it hard to interpret | Thank you for this critical observation. We have improved the figure to include the key. |
| 44 | Figure 2 has some hidden parts (large parts of it are black), it’s not clear what’s being portrayed | We have improved the figure and removed the dark parts |
| 45 | In Figure 3, what do the numbers on the x-axis represent? Also, given that week of symptom onset was recorded per individual among sampled cases, it’s not clear where the big numbers of cases on the y-axis come from | The numbers of the x-axis represent the cases per week. These are based on the 9,230 cases line listed and identified from the four health facilities which were visited in Acaba subcounty.  The epicurve is based on 9,230 cases not the sampled cases that are in the case-control study.  The case control study in an outbreak investigation is conducted after a descriptive epidemiology has been done. |
|  | **Case Control section** |  |
| 46 | Table 1: With 1:1 matching, how come you had 4 cases in the under 5 years of age and only 1 among controls in this group? Then also, 30% cases from the 5-17 years group and 37% among controls? | At design level, it was hard to get controls for anyone below 5 years who had not suffered from malaria within the stipulated time period.  We did not match by age but by age-group. We used an age-range of -/+10 matching with the cases. Hence that is the reason we have 4 cases under 5 years and 1 control because others controls are part of the 5–17-year group.  The same principle applies for the 5-17 age-group. |
| 47 | Additionally, it’s not clear what units the average net usage per household and that per week take on. Are these days or what? | We have taken note of this comment. We have deleted it to avoid confusion since we did not even include it anywhere in the discussion. |
| 48 | Table 2: Please revise this table to include the actual p-values and include the reference group for each variable indicated | We have revised accordingly as seen in the table 2. |
| 49 | Line 270 indicates engaging in activities, which activities? Also, the statement is not supported by your results given that those <100m were not shown to be associated, at least not significantly. | We have included the activities in Table 1 on page 16.  Activities within <100m were associated with infection when we did the reanalysis. |
| 50 | Line 307-309 – It’s not clear from your results that the activities within swamps increased or were different from year to year and therefore, these statements may not be supported by your results. | We have taken note of this important comment. We have modified the write up accordingly from line 401 to 405. |
| 51 | Line 315 – For inappropriate case management referred to, the evidence used is well before the duration of this study i.e. 2004, 2009 and 2012. This has certainly changed over the years and so please consider providing more relevant evidence for your claim | We have updated the write up and included more recent literature. From line 445 to 449 and from 456 to 462. |
| 52 | Lines 326-332 – This section seems to be fit for the background of your paper. It’s not clear which results are being discussed in this section. Please consider revising or moving to a more appropriate location. | Thank you for this observation. We have modified the write up accordingly. We have deleted and move d to the introduction section from line 74 to 76. |
| 53 | Line 341-342 – It’s not clear what this sentence is presenting. Simply because the report indicated (that is also not available at the reference provided) highlights the possible effects of weak surveillance doesn’t make an appropriate source of evidence of the existence of weak surveillance. | Modified the write up accordingly |
| 54 | Line 348-349 – The relevance of this sentence is also not clear. There seems to be a mix up of monitoring that was apparently done well from your statements and response from higher levels (district and MoH) which appears to have been delayed. Please make this paragraph a little more focused. | Tried to focus the write up accordingly as seen from 445 to 449 and 457 to 461. We focused to the delayed response from national level and tried to explain what exactly happened on ground during the investigation. |
| 55 | Lines 358-359 – It is surprising that your first limitation is about recording of deaths. This study you indicated was looking at confirmed malaria cases. At what point did the deaths then become a source of limitations in studying cases? | Deleted the statement on deaths as seen on line 472 to 474 on page 23 |
| 56 | Line 370 – The statement “to respond timely after …” is rather grammatically confusing, please consider revising. | Modified accordingly. |
