# Supplementary material for: Malaria outbreak facilitated by engagement in activities near swamps following increased rainfall and limited preventive measures: Oyam District, Uganda
Source: PLOS Glob Public Health. 2022 Aug 23;2(8):e0000239. doi: 10.1371/journal.pgph.0000239 (PMC10021189; doi:10.1371/journal.pgph.0000239)
Supplement: Attachment — Submitted filename: Response to Reviewers.doc [file pgph.0000239.s002.doc]

**July 1^st^, 2022**

The Editor-in-Chief

*PLOS Global Public Health*

Dear Editor,

**Re: Revised Manuscript: “Malaria Outbreak Facilitated by Engagement in Activities near Swamps Following Increased Rainfall and Limited Preventive Measures: Oyam District, Uganda, PGPH-D-21-00604”**

We are grateful for the opportunity to revise our manuscript entitled, “**Malaria Outbreak Facilitated by Engagement in Activities near Swamps Following Increased Rainfall and Limited Preventive Measures: Oyam District, Uganda**”. The reviewers’ comments were very helpful in improving the paper in a substantial manner.

On the next page, we present a point-by-point response to each of the comments raised. I should note that in the process of revising the paper, we were able to make adjustments and improvements across the paper to ensure that the paper is generally improved for your readers.

As advised, we have submitted three documents: a) a clean copy of the revised manuscript; b) a copy of the revised manuscript with track changes; and c) a point-by-point response to the comments raised (see page 2 below).

Once again, we are very grateful for the opportunity to revise the paper and we look forward to seeing the paper accepted and eventually published in your prestigious journal, PLOS Global Public Health.

Yours sincerely,

Maureen Katusiime

**Corresponding author**

**POINT-BY-POINT RESPONSE TO REVIEWERS’ COMMENTS**

| **No** | **Comment** | **Response** |
| --- | --- | --- |
|  | **Reviewer Comments** |  |
|  | **Reviewer 1** |  |
| 1 | In line 30, I suggest remove vector behavior, since in your methods and results, it is not captured.  I think “ to determine vector density and potential aquatic Anopheles habitats” clear states what you did. | We think this is an important observation that was raised by the reviewer. We have modified the sentence and deleted the word “behavior”. |
| 2 | In line 43, “ scoop+”, do you mean positive for larvae? | We think this is an important observation that was raised by the reviewer. This was a typing error and have deleted the “+” sign. |
| 3 | In line 47, use conservative statement like, “This outbreak was likely facilitated by Anopheles aquatic habitats near homes” | We have taken note of this important suggestion and included “likely” in the sentence. |
| 4 | From line 178-183, seems to state that all the aquatic habitats sampled were containers | We thank you for this comment. We have modified the writeup and moved mosquito larval assessment subsection to line 173 from line 181 because we used larval scoops described from line 175 to line 179 as well as the household and container indices as to determine larval density. |
| 5 | Why do you need household index and container index? In addition, you can’t say houses are positive for larvae because we know larvae are found in aquatic habitats | We have taken of the question. In line 189 to 191, we give reasons why we used household and larval index.  In line 184 to 185, we have modified the formula to reflect number of houses infested/number of houses inspected as captured by WHO |
| 6 | To compute larval density, I would simply calculate average larvae per dip and express it as average larvae per dip per habitat type.  The aim would be to know which habitat type is most productive for Anopheles. If you did not record number of dips per habitat during data collection, then it’s difficult, you could record the number of larvae per habitat type although this depends on sampling effect. | We thank you for this suggestion. Much as we would like to adopt it, we remain limited because we did not count larvae per container even when larvae were counted for the other aquatic habits. |
| 7 | In line 268, “we found a parity rate of 14% and longevity of 2.1 days”, You mean on average mosquitoes live for 2 days. Line 275-276 need to be changed if you agree with my comments on calculating larval density. | Yes that is what our study found out. According to WHO, there are some mosquitoes with such a longevity.  We appreciate the proposed suggestion by the reviewer but request that we maintain the results from the methods used much as we have made some modification from line 280 to line 287. |
| 8 | In line 313, the statement “Notably, 20% of mosquitoes captured in homes were male, potentially representing a very high larval density” may not be accurate. It implies male mosquitoes are an indicator of larval density, yet in the methods you calculated larval density. | We have modified the statement accordingly on line 326-327 to cater for the comment raised. |
| 9 | Correct the error in line 331 “it is” | We have modified the sentence and separated the words |
| 10 | In line 335, see my comments on container and household index? | This was noted although it was not very clear. We tried to make a few adjustments. |
|  | **Reviewer 2** |  |
| 11 | Line ♯ 69-70: The sentence is not quite clear | We thank you for this observation. This has been addressed on line 60. We modified the sentence to include “ that the country still has” to make the statement clearer. |
| 12 | Line ♯ 72: It is not clear here. Do you mean good data quality, completeness, and ability to analyze data compromise the adequacy of malaria surveillance system? This would be misleading to the reader | We have modified the sentence to have negative attributes on line 72 and 73. We changed having an “having an adequate malaria surveillance system” to “an inadequate”; included poor on data quality, changed complete to incomplete as shown in the sentence. |
| 13 | Line ♯ 91: How is it that you conducted an outbreak? This is rather confusing | This has been addressed and included outbreak investigation |
| 14 | Line ♯ 319-320: The Sentence is not quite clear and in a couple other places this occurs where words are unclear because there’s no space between what appears to be the right separate words. | This has been addressed accordingly |
| 15 | Line ♯ 384-385: The sentence that starts with “ A decline in cases …..\|” is not clear | We have modified to include “at” on line 401 |
| 16 | Line ♯ 400: It’s not at all clear what your recommendation of “ mass case management…” means in real life | This has been addressed on line 416 and 417. We have replaced “mass case management” with “mass drug administration of antimalarial treatment to residents in Acaba Subcounty” |
|  | **Journal Requirements** |  |
| 17 | Please review your reference list to ensure that it is complete and correct. If you have cited papers that have been retracted, please include the rationale for doing so in the manuscript test, or remove these references and replace them with relevant current references. Any changes to the reference list should be mentioned in the rebuttal letter that accompanies your revised manuscript. If you need to cite a retracted article, indicate the article’s retracted status in the Reference list and also include a citation and full reference for the retraction notice. | We thank you for this guidance. We have reviewed the list, it is complete and correct.  We have not cited papers that have been retracted.  We have removed 8 some references from list and replaced them with more recent and relevant ones.  **Below are the references removed:**   1. Ministry of Health, Health Systems 20/20, and Makerere University School of Public, Health. Uganda Health System Assessment 2011 [Internet]. Kampala, Uganda and Bethesda: Health Systems 20/20 project, Abt Associates Inc; 2012 Apr p. 85–91. Available from: <https://health.go.ug/docs/hsa.pdf> 2. Yeka A, Gasasira A, Mpimbaza A, Achan J, Nankabirwa J, Nsobya S, et al. Malaria in Uganda: Challenges to control on the long road to elimination. Acta Trop. 2012 Mar;121(3):184–95. 3. USAID PRESIDENT’S MALARIA INITIATIVE. Uganda - Malaria Operational Plan FY 2019 [Internet]. Uganda; [cited 2021 Aug 10] p. 82. Available from: https://dc.sourceafrica.net/documents/119356-Fy-2019-Uganda-Malaria-Operational-Plan.html 4. Ministry of Health. THE UGANDA MALARIA REDUCTION AND ELIMINATION STRATEGIC PLAN 2021-2025. Ministry of Health; 2020. 5. Joyce Nguna, Dennis Okethwangu and Daniel Kadobera. Malaria Outbreak Facilitated by Increased Human Activities around Swamps: Kisoro District, Uganda, December 2017-March 2018. 2019 Jul; Available from: <https://www.researchgate.net/publication/340208827> 6. Irene Mirembe. To Truly Manage Malaria in Uganda, Diagnosis Matters. 2018 Apr 14; Available from: <https://www.intrahealth.org/vital/truly-manage-malaria-uganda-diagnosis-matters> 7. Arrow KJ, Panosian C, Gelband H, editors. Saving Lives, Buying Time: Economics of Malaria Drugs in an Age of Resistance [Internet]. Washington (DC): National Academies Press (US); 2004.; 2004. Available from: <https://www.ncbi.nlm.nih.gov/books/NBK215619> 8. Lukwago L, Nanyunja M, Ndayimirije N, Wamala J, Malimbo M, Mbabazi W, et al. The implementation of Integrated Disease Surveillance and Response in Uganda: a review of progress and challenges between 2001 and 2007. Health Policy Plan. 2013 Jan 1;28(1):30–40. 9. Catherine Ntabadde Makumbi. “Test and Treat” strategy reinforced in the implementation of UKaid’s Malaria Programme. Unicef uganda [Internet]. 2019 Apr 12; Available from: https://www.unicef.org/uganda/stories/test-and-treat-strategy-reinforced-implementation-ukaids-malaria-programme   **New references include:**   1. Oguttu DW, Matovu JKB, Okumu DC, Ario AR, Okullo AE, Opigo J, et al. Rapid reduction of malaria following introduction of vector control interventions in Tororo District, Uganda: a descriptive study. Malar J. 2017 Dec;16(1):227. 2. PRESIDENT’S MALARIA INITIATIVE. Uganda Malaria Operational Plan FY 2021 [Internet]. 26 p. Available from: <https://d1u4sg1s9ptc4z.cloudfront.net/uploads/2021/03/fy-2021-uganda.pdf> 3. Ministry of Health. National Malaria Control Program: Malaria Bulletin 2016. 1(1). Available from: <https://www.health.go.ug/cause/malaria-bulletin-2016/> 4. Okiring J, Routledge I, Epstein A, Namuganga JF, Kamya EV, Obeng-Amoako GO, et al. Associations between environmental covariates and temporal changes in malaria incidence in high transmission settings of Uganda: a distributed lag nonlinear analysis. BMC Public Health. 2021 Dec;21(1):1962. 5. Siya A, Kalule BJ, Ssentongo B, Lukwa AT, Egeru A. Malaria patterns across altitudinal zones of Mount Elgon following intensified control and prevention programs in Uganda. BMC Infect Dis. 2020 Jun 17;20(1):425. 6. Ministry of Health. National Technical Guidelines for Integrated Disease Surveillance and Response: Third Edition [Internet]. 2021. Available from: <https://www.afro.who.int/sites/default/files/2021-09/2> 7. Wang H, Wang Y, Cheng P, Wang H, Wang H, Liu H, et al. The Larval Density of Mosquitos (Diptera: Culicidae) in Jiaxiang County, Shandong Province, China: Influence of Bacterial Diversity, Richness, and Physicochemical Factors. Front Ecol Evol [Internet]. 2021;9. Available from: https://www.frontiersin.org/article/10.3389/fevo.2021.616769   **Modified references include:**   1. Simple O, Mindra A, Obai G, Ovuga E, Odongo-Aginya EI. Influence of Climatic Factors on Malaria Epidemic in Gulu District, Northern Uganda: A 10-Year Retrospective Study. Malar Res Treat. 2018 Aug 13;2018:5482136. 2. Uganda Ministry of Health. The Uganda Malaria Reduction and Elimination Strategic Plan 2021-2025. 2020. 3. Global Partnership to Roll Back Malaria. A framework for field research in Africa : malaria early warning systems : concepts, indicators and partners. [Internet]. World Health Organization; 2001. Available from: <https://apps.who.int/iris/handle/10665/66848> 4. World Health Organization. Division of Malaria and other Parasitic Diseases. Manual on practical entomology in malaria [Internet]. World Health Organization; Available from: <http://apps.who.int/iris/handle/10665/42481> 5. Barbosa LMC, Souto RNP, dos Anjos Ferreira RM, Scarpassa VM. Behavioral patterns, parity rate and natural infection analysis in anopheline species involved in the transmission of malaria in the northeastern Brazilian Amazon region. Acta Trop. 2016 Dec 1;164:216–25. 6. World Health Organization, editor. Comprehensive guidelines for prevention and control of dengue and dengue haemorrhagic fever [Internet]. Rev. and expanded. ed. New Delhi, India: World Health Organization Regional Office for South-East Asia; 2011. 196 p. (SEARO Technical publication series). Available from: http://apps.who.int/iris/bitstream/handle/10665/204894/B4751.pdf?sequence   Others have remained as previously shared. |
